# Commentary on ‘Oldhamite: a new link in upper mantle for C-O-S-Ca cycles and an indicator for planetary habitability’

**DOI:** 10.1093/nsr/nwad185

**Published:** 2023-06-28

**Authors:** Fabrizio Nestola

**Affiliations:** Department of Geosciences, University of Padova, Italy

The paper by Liu *et al.* [1] is aimed to discuss the role of oldhamite (CaS) in the understanding of the C-O-S-Ca cycles of the Earth. The results of this manuscript also demonstrate the meaning of the presence of oldhamite on the surface and interior of a planet by taking a view of its stability on Earth as well as on other bodies in the Solar System.

Oldhamite (*Fm*-3 *m, a*  =  5.69 Å with V/10^6^ pm^3^ = 184.88) is stable in highly reducing conditions with an oxygen fugacity below IW + 3.3 (IW = iron-wüstite redox buffer, in *lgf*o_2_) [1]. In addition, it was demonstrated that this rare mineral is stable at temperatures greater than 700°C [2] (thermal expansion coefficient of 4.03 × 10^−5^ K^−1^). I agree with the authors that oldhamite could have been involved as a transient phase in important geological processes [1] as the examples reported by them: i) the mantle metasomatism by carbonate melts beneath the MORB region and ii) the crustal calcite contamination of mantle-derived magma during the formation of some magmatic Cu-Ni-PGE sulfide deposits. The possibility to consider CaS as a transient phase as a function of oxygen fugacity opens new scenarios on deep geological processes where S is involved and we cannot exclude the presence of further rare sulfides both within the Earth and on other Planetary bodies. Indeed, even if locally, it was shown that oxygen fugacity within the Earth could be extremely variable [3] and thus the mineralogical, petrological and geochemical community should start to take into consideration what is thought to be rare minerals, like CaS and other sulfides.

CaS is extremely rare on Earth and only two studies reported the potential occurrence of this mineral in natural terrestrial rocks (e.g. on volcanic glass and on an impactite [4,5]). This sulfide is well-known in meteorites (e.g. enstatite chondrites, aubrites, lunar meteorites [1], and ungrouped achondrites [6]) and as one of the major components of hollows and pits on the surface of Mercury [2,7]. However, even if I agree that investigating the role of CaS is crucial for the understanding of the C-O-S-Ca cycles, at the same time, it would be of interest to conduct similar experiments on niningerite, MgS. Indeed, in meteorites (enstatite chondrites), as well as in the pits and hollows of Mercury, oldhamite is always associated with the presence of niningerite [8,9]. In addition, as the partial melts from orthopyroxene in their experiments are basaltic melts or high-Mg basaltic melts, it could be relevant to consider the presence of this phase and to estimate oxygen fugacity for its geological stability. The mineral physics investigation of MgS and CaS (and likely other sulfides abundant in meteorites [10]), will be useful in understanding the formation of these phases (as transient phases) from S-fluids at different P-T regimes in the interiors of our planet and to understand geological information on the surfaces of other planetary bodies.

***Conflict of interest statement*.** None declared.
